# Editorial: Micro/nanorobots in nanobiotechnology

**DOI:** 10.3389/fbioe.2024.1453307

**Published:** 2024-07-10

**Authors:** Fengtong Ji, Tianlong Li, Katherine Villa, Yue Dong

**Affiliations:** ^1^ Wellcome Trust/Cancer Research UK Gurdon Institute, University of Cambridge, Cambridge, United Kingdom; ^2^ Department of Physiology, Development and Neuroscience, University of Cambridge, Cambridge, United Kingdom; ^3^ State Key Laboratory of Robotics and System, Harbin Institute of Technology, Harbin, China; ^4^ Institute of Chemical Research of Catalonia (ICIQ), The Barcelona Institute of Science and Technology (BIST), Tarragona, Spain; ^5^ Guangdong Provincial Key Laboratory of Intelligent Morphing Mechanisms and Adaptive Robotics, School of Mechanical Engineering and Automation, Harbin Institute of Technology, Shenzhen, China

**Keywords:** nanotechnology, micro/nanorobots, biomedicine, cancer therapy, clinical treatment, medical diagnosis

Six decades have passed since Richard Feynman spearheaded one research direction towards small-scale machinery, heralding the dawn of nanotechnology. Advances in microscopy have significantly enhanced our understanding of fundamental principles at the microscopic level and have provided us with versatile methods for control in microenvironments. Among these advancements, micro/nanorobots emerge as unique devices that facilitate direct interaction with microscopic entities, such as cells and molecules. These tiny machines, powered by field energy conversion, enable precise control and serve as customisable building blocks to perform tasks in various environments, particularly within complex biological systems. This Research Topic aims to elucidate nanorobot-cell interactions and inspire the development of novel functions for micro/nanorobots in biological contexts, ultimately advancing innovative treatments in clinical applications.

Considering the potential of micro/nanorobots in the biomedical field, the review articles in this Research Topic offer insights into the capabilities of such self-propelled systems in medicine. Active control of micro/nanorobots can enhance local drug delivery and improve therapeutic effectiveness whilst minimising side effects on healthy tissues. Passive nanomedicines or non-motile particles have proven effective in cancerous environments, including tumours in the liver, breast, glioma, pancreas, lung, oesophagus, and cervical regions, as well as in treating endocrine diseases such as diabetic complications and circulatory system diseases, such as ischemic stroke and thrombosis. Integrating nanorobotics into these systems has the potential to synergistically improve targeted drug delivery, biological barrier penetration (such as the blood-brain barrier and deep tissues), and overall treatment efficiency.

Nanorobots can facilitate tissue growth and organoid formation when we examine their interactions with tissues. At the cellular level, they enhance intracellular delivery of substances such as oxygen (Zhang et al., 2019), siRNA (Esteban-Fernández De Ávila et al., 2016), and other complexes, thereby achieving gene silencing (Yin et al., 2013) and knockout (Hansen-Bruhn et al., 2018). Both the cytoplasm and nucleus are accessible targets (He et al., 2022). The hydrogel magnetic microrobots discussed in this Research Topic have been employed to deliver drugs that inhibit cancer cell proliferation and mobility and induce apoptosis.

In the realm of biomedical diagnosis, functionalised micro/nanorobots can bind to target genes, activate fluorescence signals, and enable rapid and sensitive diagnoses for conditions such as head and neck cancer (Qualliotine et al., 2019), HeLa cells (Esteban-Fernández De Ávila et al., 2015), and Alzheimer’s disease (Sun et al., 2021). They can also detect the SARS-CoV-2 virus by recognising its spike protein (Mayorga-Martinez et al., 2022). Additionally, nanorobots can enhance the efficiency of the enzyme-linked immunosorbent assay (ELISA) (Wang et al., 2022) and improve imaging diagnoses of deep tissues, such as intestinal diseases, which are traditionally examined using an endoscope.

A typical treatment cycle of a nanorobotic system comprises four sections: drug loading, targeted drug delivery, drug release (if the drugs are coated internally or require specific contact with tissues for efficacy), and nanorobot degradation or exclusion from the body. Regarding actuation, three external driving fields are discussed in this Research Topic: acoustic, magnetic, and electrical fields, due to their biocompatibility and non-contact control manners. Additionally, light, thermal and chemical (Simó et al., 2024) energies may enhance some objectives, endowing nanorobots with multiple functions to navigate complex and dynamic environments. Biosafety is a priority when applying micro/nanorobots in biological environments and during imaging procedures such as positron emission tomography (PET), computed tomography (CT), photoacoustic computed tomography (PACT), optical coherence tomography (OCT), magnetic resonance imaging (MRI), and X-ray. A potential solution is to integrate living cells (e.g., microorganisms, macrophages, platelets, stem cells, and chimeric antigen receptor T cells) or cell membranes (e.g., platelet, tumour cell, macrophage, and red blood cell membranes) to fabricate biohybrid micro/nanorobots. These have proven effective in modulating the immune system (Zhou et al., 2020).

Numerous reviews have emerged during COVID-19 and its aftermath due to the lockdown, but now most research activities, including experiments, have returned to normal thanks to the sacrifices made by all humanity. Clinical trials and medical translation of nanobiotechnology are complex processes that take time to manage. Biological experiments demand significant time commitment and patience to obtain valid data proving efficacy. Long-term dedication is essential for researchers to solidify the fundamentals, and cross-disciplinary integration is necessary to bridge the gap between laboratory research and clinical applications. Biomimetic inspiration can be drawn from nature for materials and designs, meanwhile, emerging technologies including artificial intelligence (Yang et al., 2024) may drastically advance nanorobotic biotechnology in healthcare. Nowadays, open-access articles are becoming a new trend removing barriers to knowledge, and the impact of refereed articles relies on researchers’ integrity and scientific discoveries. Time will reveal what research is pivotal and constructive to the community and society, guiding younger generations to shape the future together. We look forward to witnessing the breakthroughs of nanorobots that address the health challenges everyone faces.
